# Walking as a Contributor to Physical Activity in Healthy Older Adults: 2 Week Longitudinal Study Using Accelerometry and the Doubly Labeled Water Method

**DOI:** 10.2196/mhealth.5445

**Published:** 2016-06-07

**Authors:** Giulio Valenti, Alberto G Bonomi, Klaas R Westerterp

**Affiliations:** ^1^Department of Human BiologyMaastricht UniversityMaastricht, 6200 MDNetherlands; ^2^Personal Health SolutionsPhilips Research LaboratoriesEindhovenNetherlands; ^3^Department of Human BiologyMaastricht UniversityMaastrichtNetherlands

**Keywords:** aging, walking, physical activity, accelerometry, monitoring, ambulatory/instrumentation

## Abstract

**Background:**

Physical activity is recommended to promote healthy aging. Defining the importance of activities such as walking in achieving higher levels of physical activity might provide indications for interventions.

**Objective:**

To describe the importance of walking in achieving higher levels of physical activity in older adults.

**Methods:**

The study included 42 healthy subjects aged between 51 and 84 years (mean body mass index 25.6 kg/m^2^ [SD 2.6]). Physical activity, walking, and nonwalking activity were monitored with an accelerometer for 2 weeks. Physical activity was quantified by accelerometer-derived activity counts. An algorithm based on template matching and signal power was developed to classify activity counts into nonwalking counts, short walk counts, and long walk counts. Additionally, in a subgroup of 31 subjects energy expenditure was measured using doubly labeled water to derive physical activity level (PAL).

**Results:**

Subjects had a mean PAL of 1.84 (SD 0.19, range 1.43-2.36). About 20% of the activity time (21% [SD 8]) was spent walking, which accounted for about 40% of the total counts (43% [SD 11]). Short bouts composed 83% (SD 9) of walking time, providing 81% (SD 11) of walking counts. A stepwise regression model to predict PAL included nonwalking counts and short walk counts, explaining 58% of the variance of PAL (standard error of the estimate=0.12). Walking activities produced more counts per minute than nonwalking activities (*P*<.001). Long walks produced more counts per minute than short walks (*P*=.001). Nonwalking counts were independent of walking counts (*r*=−.05, *P*=.38).

**Conclusions:**

Walking activities are a major contributor to physical activity in older adults. Walking activities occur at higher intensities than nonwalking activities, which might prevent individuals from engaging in more walking activity. Finally, subjects who engage in more walking activities do not tend to compensate by limiting nonwalking activities.

**Trial Registration:**

ClinicalTrials.gov NCT01609764; https://clinicaltrials.gov/ct2/show/NCT01609764 (Archived by WebCite at http://www.webcitation.org/6grls0wAp)

## Introduction

Aging is accompanied by reduced physical activity (PA) and increased sedentary behavior [1-3]. The time spent by older adults in moderate to vigorous PA decreases from 30 minutes/day in individuals in their seventh decade to 10 minutes/day in individuals older than 80 years [4]. Reduced PA is associated with reduced mobility [5] and decreased life expectancy [6,7]. To contrast this trend, the World Health Organization guidelines suggest that subjects older than 65 years should be moderately active for at least 150 minutes/week in bouts longer than 10 minutes [8]. Although a variety of activities are carried out during the day, it is unknown which one is more effective in increasing PA.

Among the activities of daily living, walking is of major importance. Walking is an indicator of overall well-being and walking speed is an indicator of life expectancy. Independent living strongly depends on walking, which allows individuals to accomplish many tasks of daily living [9]. Furthermore, walking is a highly prevalent form of PA in healthy older individuals, who reportedly walk as much as when they were younger and more physically active [10]. In spite of its relevance, the role of walking in PA has not yet been described and it is unclear whether active people actually walk more. Individuals who frequently engage in walking activities might compensate by decreasing nonwalking activities, such as biking, practicing sports, housekeeping, or climbing stairs, ultimately resulting in similar levels of PA as individuals less inclined to walk. The definition of the role of walking in PA might provide more activity-specific insights as well as intervention indications.

Quantification of walking activity can be provided by inertial sensors such as pedometers [11,12] or accelerometers [13,14]. Both pedometers and accelerometers offer the possibility to monitor walking activities over time. Pedometers only measure number of steps, whereas more information, including nonwalking activities, might be derived from accelerometers. To date, studies where walking patterns are described by means of an accelerometer have not integrated the results of overall PA, leaving out the effects of nonwalking activities [15,16]. This study aimed at describing the importance of walking in achieving higher levels of PA, as measured using doubly labeled water in older adults.

## Methods

### Population

The population included 35 subjects from a previous study and 7 subjects with similar characteristics [17]. All 42 healthy subjects (19 males and 23 females) aged between 51 and 84 years (mean body mass index 25.6 kg/m^2^ [SD 2.6]) were recruited by advertisements in local newspapers (Table 1). After signing a written informed consent form, respondents completed a questionnaire including information regarding orthopedic conditions, neurological disorders, and cardiovascular problems that could affect the study. The questionnaire was discussed during a medical visit with a doctor. All subjects were in good orthopedic, neurological, and cardiovascular health and were therefore included. The study was conducted according to the Declaration of Helsinki, and the Ethics Committee of the Maastricht University Medical Center approved the study. This trial was registered at ClinicalTrials.gov as NCT01609764.

**Table 1:** Subject characteristics (N=42, 19 males).

Characteristic	Mean (SD)	Range
Age, years	65 (8)	51-84
Height, m	1.69 (0.10)	1.47-1.89
Body mass, kg	73 (11)	40-95
BMI^a^, kg/m^2^	25.6 (2.6)	18.6-30.0

^a^ BMI: body mass index.

### Study Design

Physical activity, walking, and nonwalking activity were monitored with an accelerometer for 2 weeks. Additionally, total energy expenditure (TEE) and basal metabolic rate (BMR) were measured in a representative subgroup of 31 randomly selected subjects. Physical activity level (PAL) was then calculated as the ratio of TEE to BMR [18].

### Walking Recognition Algorithm

Subjects wore a triaxial accelerometer (GT3X+, ActiGraph, Pensacola, FL) on the lower back using a belt, as described before [18]. Accelerometry data were collected in the laboratory and in daily life at a sampling rate of 60 Hz. During the laboratory session, subjects performed treadmill walking at 4 different speeds. Such data were used to define parameters of the algorithm used to detect walking activities. A personalized template prototype of the acceleration signal was derived from the treadmill data for each subject, as described before [17]. Accelerometer data were analyzed as vector magnitude and segmented into epochs of 5 seconds. The acceleration signal from each epoch was cross-correlated with the personalized template. The standard deviation of the acceleration signal (SDs) and the standard deviation of the cross-correlation function output (SDcc) were calculated for all epochs. SDs and SDcc from treadmill epochs were used to fit two probability distributions indicating the likelihood of walking. A Naïve Bayes probability distribution for the laboratory walking data was defined as follows:

      P(walk) = P(walk | SD_s_ ∧ SD_cc_) (1)

      P(nonWalk)=1−P(walk) (2)

Assuming naïve conditional independence,

     P(walk) = *k* × P(SD_s_ | walk) × P(SD_cc_ | walk) (3)

with

      *k* = P(walk)/P(SD_s_ ∧ SD_cc_) (4)

P(SD_s_ | walk) and P(SD_cc_ | walk) were derived from the data and k was empirically estimated as follows:

     *k* = 0.5/(0.14 × max(P(SD_s_ ∧ SD_cc_ | walk))) (5)

The classifier for each epoch in daily life was constructed as follows:

      Y=argmax (P(walk), P(non-walk)) (6)

Bouts of walking shorter than 1 minute were classified as short walks, whereas bouts lasting at least 1 minute were classified as long walks.

Counts were calculated by integrating body accelerations of each epoch after detrending and rectification of the signal from each axis. Epochs with less than 10^-3^ counts/second were labeled as inactivity and excluded from further analysis.

Nonwalking counts, short walk counts, and long walk counts per day were calculated integrating counts in the respective epochs over each day. Walking counts were the sum of short walk counts and long walk counts. Activity counts were the sum of walking counts and nonwalking counts. Time spent in each category was measured as 5 seconds multiplied by the respective number of epochs. Days during which data were missing or subjects carried the accelerometer for less than 10 hours were excluded and the average was calculated for the remaining data, assuming that daily PA is an ergodic process where the expected mean does not change after removing a randomly taken sample. Average counts per day were calculated over the days of measurement.

### Energy Expenditure

Total energy expenditure was measured during the 2 weeks of measurement using doubly labeled water, according to the Maastricht protocol [19]. Briefly, after the collection of a baseline urine sample on the evening of day 0, subjects drank a weighted amount of ^2^H_2_^18^O. The result is an initial increase in the body water enrichment of about 120 ppm for ^2^H and about 240 ppm for ^18^O. Urine samples were then collected in the morning (from the second voiding) of days 1, 8, and 15 and in the evening of days 1, 7, and 14. Samples were analyzed by isotope ratio mass spectrometry (Optima; VG Isogas, Middlewich, Cheshire, UK). Carbon dioxide production was calculated from the difference between the elimination rates of ^2^H and ^18^O. Total daily energy expenditure was calculated from carbon dioxide production [20], assuming a respiratory quotient of 0.85. Basal metabolic rate was measured during 30 minutes under a ventilated hood (Omnical; Maastricht Instruments, Maastricht, the Netherlands) on the morning of the first day of measurement in the supine position under standard conditions of rest, fasting, immobility, thermoneutrality, and mental relaxation.

### Data Analysis

All variables are expressed as mean (SD). The normality of the data was examined with the Shapiro-Wilk test. The Pearson correlation coefficient (*r*) was used to describe the association between variables. A linear regression was used to model the relation between activity counts and PAL. The residuals of the model were studied to identify confounding factors. The best predictors of PAL among nonwalking counts, short walk counts, and long walk counts were selected using a stepwise multilinear regression. The statistical significance threshold was set at *P*<.05. MATLAB (MathWorks Inc, Natick, MA, USA) was used for the data elaboration and the figures. SPSS (SPSS Inc, Chicago, IL, USA) was used for the statistical analysis.

## Results

**Figure 2:**
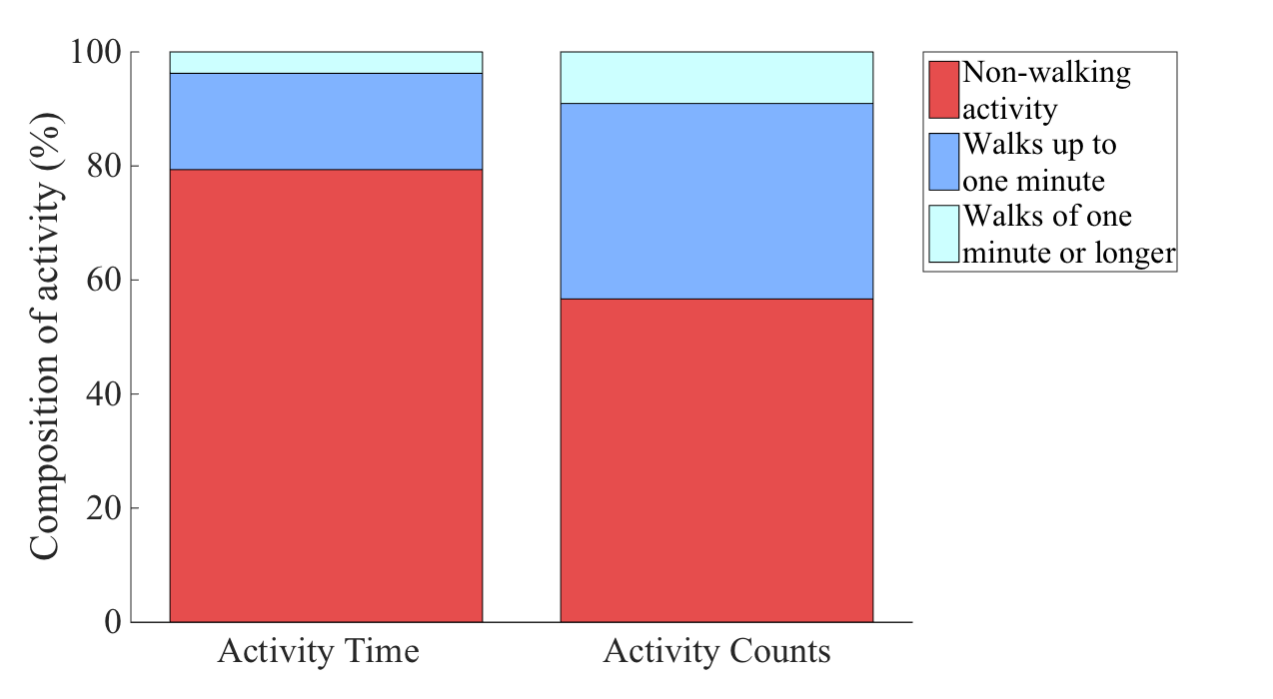
Composition of activity time and activity counts in older adults (N=42).

Physical activity level ranged between 1.43 and 2.36 (1.84 [SD 0.19] on average, N=31). The activity time of the total group (N=42) was on average 10.5 hours/day (SD 1.7). About 20% of the activity time (21% [SD 8]) was spent in walking activities, which accounted for about 40% of the total counts (43% [SD 11]; Figure 2). The average walking time was about 2 hours/day, indicating that PA was not restricted in this population. Subjects walked in short bouts for 83% (SD 9) of their walking time providing 81% (SD 11) of their walking counts (Table 2).

Higher PAL was achieved by subjects with higher activity time (*r*=.75, *P*<.001) and activity counts (*r*=.73, *P*<.001). Specifically, subjects with a higher PAL spent more time in nonwalking activities (*r*=.64, *P*<.001) and produced more nonwalking counts (*r*=.61, *P*<.001) than subjects with a lower PAL. The correlation coefficient between PAL and walking, particularly during short walks, was positive but did not reach significance (PAL vs walking time: *r*=.26, *P*=.08; PAL vs walking counts: *r*=.33, *P*=.07; PAL vs short walk counts: *r*=.35, *P*=.05).

Two linear models were developed to identify the best predictors of PAL. A simple linear model based on activity counts could explain 52% of the measured PAL (standard error of the estimate [SEE]=0.13). The residuals of this model correlated with the fraction of walking time spent in short walks (*r*=−.38, *P*=.02), suggesting that the prevalence of short walks might be a determinant of PAL. Therefore, a stepwise regression model was developed to predict PAL from one or more variables among nonwalking counts, short walk counts, and long walk counts (Table 3). This second model included nonwalking counts and short walk counts, explaining 58% of the variance of PAL (SEE=0.12).

**Figure 3:**
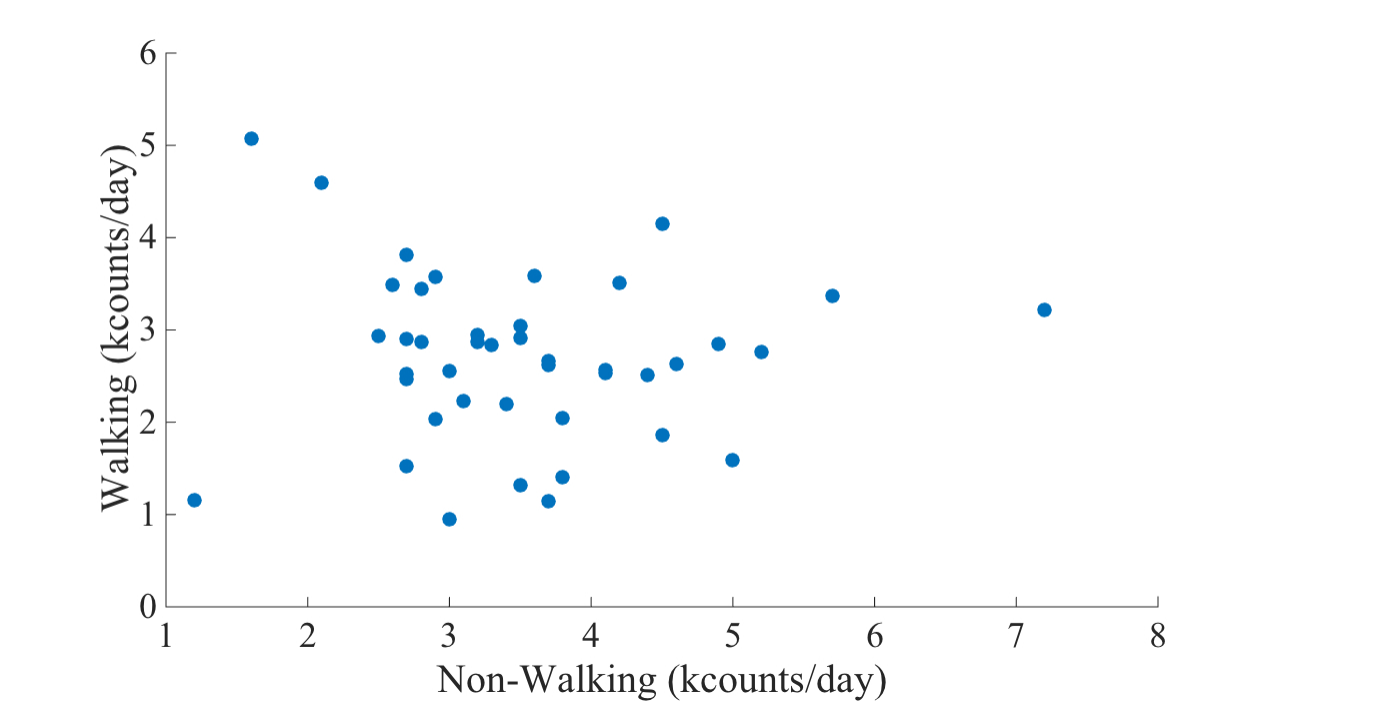
Walking counts versus nonwalking counts in older subjects (N=42).

Walking activities were conducted at higher intensities than nonwalking activities (mean 21.5 [SD 3.0] counts/minute vs mean 7.0 [SD 1.2] counts/minute, *P*<.001). Among walking activities, long walks were conducted at higher intensities than short walks (mean 23.3 [SD 3.6] counts/minute vs mean 20.9 [SD 3.1] counts/minute, *P*=.001). Walking counts were independent of nonwalking counts (*r*=−0.05, *P*=.38; Figure 3).

**Table 2:** Physical activity and walking activity (N=42); mean (SD).

Variable	Time (minutes/day)	Counts (kcounts/day)	Intensity (counts/minute)
Physical activity	630 (101)	6.2 (1.4)	9.8 (1.2)
Nonwalking activity	500 (97)	3.5 (1.1)	7.0 (1.2)
Walking activity	130 (61)	2.7 (0.9)	21.5 (3.0)
Walks <1 minute	106 (49)	2.1 (0.7)	20.9 (3.1)
Walks >1 minute	24 (17)	0.6 (0.4)	23.3 (3.6)

**Table 3:** Two prediction equations of physical activity level from accelerometer counts.

Model	Variable^a^	Coefficient	*P* ^b^	*r* ^2c^	SD^d^	Beta^e^
Linear model						
	Intercept	1.24	<.001		0.11	
	Physical activity	9.70×10^−5^	<.001	0.53	1.71×10^−5^	0.73
	Model			0.53		
Multiple linear model						
	Intercept	1.17	<.001		0.11	
	Nonwalking activity	10.92×10^−5^	<.001	0.38	1.99×10^−5^	0.68
	Walks <1 minute	13.42×10^−5^	.001	0.20	3.64×10^−5^	0.46
	Model			0.58		

^a^ Variables included in the model.

^b^ Significance level.

^c^ Coefficient of determination.

^d^ Standard deviation of the coefficient.

^e^ Standardized coefficient.

##  Discussion

This is the first study that combines accelerometry and the doubly labeled water method to show that walking activities are a major contributor to PA in a population of older adults with a wide range of PAL.

The range of PAL of the subjects in this study is higher than normally reported in healthy older adults [21-23]. The activity time, including all levels of PA, was 10.5 hours/day, which is lower than previous studies where sedentary, moderate, and vigorous activities were reported to add up to 14 hours/day [24], when measured with an accelerometer in older adults. Although different, the results are compatible as in previous studies sedentary time also included periods of inactivity. This study instead excluded inactivity time to focus on PA and in particular on walking.

Walking was a main component of PA providing more than 40% of the activity counts. Despite the relevant proportion of counts, walking time was about 20% of activity time or 2 hours/day. Previous studies reported similar lengths of time spent walking in healthy and independently living older adults [25]. The relevance of walking in daily life is well established [26,27], and walking activity measured with pedometers is often used as a proxy of PA [28,29]. To our knowledge, no study has assessed walking activity as counts so far, preferring number of steps. The number of steps is not comparable to activity counts, whereas the assessment of walking activity as walking counts described in this study allowed to describe the contribution of walking to activity counts. The results showed that walking counts are a relevant proportion of activity counts, although walking time was a relatively small portion of activity time. The short amount of time spent walking is possibly the reason why walking counts only showed a trend with PAL but did not reach significance. Although walking does not seem to explain variations in PAL, its contribution in terms of counts produced remains a major one.

The discrepancy between the proportion of walking counts and the proportion of walking time implies that walking activities occurred at higher intensities and produced more counts per unit of time, compared with nonwalking activities. Walking counts have not been reported before, but previous studies reported that during walking older adults reach 80% of their maximum heart rate and oxygen consumption, concluding that walking intensity in this population is moderate to high [30]. Similarly, it can be observed that long walks were performed at higher intensities than short walks, but over a shorter portion of time. It seems therefore that older adults prefer low-intensity activities and that the high intensity discourages them from engaging in walking and, in particular, long walks. This conclusion is supported by previous studies where walking time in daily life was shown to decrease with increasing intensity [25].

Subjects who engaged in more walking activities did not compensate by reducing nonwalking activities. Although previous studies suggest that PA interventions could suffer from compensatory behaviors outside the exercise time [31-33], these results show that walking is independent of nonwalking. It is therefore reasonable to hypothesize that walking could be less prone to compensatory behaviors and therefore a good candidate for PA interventions.

There is ongoing discussion about whether PA interventions should focus on long walks or short walks [34-36]. The results of this study suggest that subjects tend to prefer shorter walks because of their relatively lower intensity. Short walks also contribute to explaining the variation in free-living PAL and therefore subjects who engaged in more short walks showed higher PAL. These might suggest that short walk interventions could be better tolerated by older individuals and effectively increase their PAL.

The ability of accelerometers to differentiate between activities allowed the detection of walking in this study. Other activities, such as biking, swimming, rowing, deskwork, housekeeping, or driving, have different acceleration patterns and were classified as nonwalking activities. Algorithms to detect these activities have been described before [37-40] and they could be used to divide the nonwalking category into more specific categories. Given that nonwalking counts partly explained the inter-individual variance in PAL, the detection of different nonwalking activities can provide a more elaborate insight into the composition and determinants of PA.

The population in this study was representative of an active and healthy older population. The findings cannot be generalized to populations with different characteristics without specific studies. Similar protocols could be used in populations that might benefit from increased PA such as obese individuals, diabetic patients, or white-collar workers. Interventions focused on walking in these populations might be effective, but more research is needed to quantify possible compensatory behaviors and to investigate other factors that can contribute to PAL, such as environmental factors, socioeconomic status, and health status.

This study provides reference values for the contribution of walking to PA in healthy older adults, as measured with wearable devices. One possible application of eHealth is in the promotion of PA. A wearable device can reveal subjects’ proportion of walking in daily life and provide them with motivational feedbacks.

The results of a previous study suggested that subjects with different walking economy produce different acceleration outputs [17]. This might have implications on the relationship between PAL and activity counts. Future studies might reveal whether walking economy can improve the prediction of PAL with an accelerometer.

In conclusion, walking activities are a major contributor to PA in older subjects with a wide range of PAL, but the relatively high intensity of walking might prevent individuals from engaging in more walking activity. It is also shown that subjects who engage in more walking activities do not tend to compensate by limiting nonwalking activities.

## Abbreviations

BMR: basal metabolic rate
PA: physical activity
PAL: physical activity level
SEE: standard error of the estimate
TEE: total energy expenditure
